# Tracking of fruit, vegetables and unhealthy snacks consumption from childhood to adulthood (15 year period): does exposure to a free school fruit programme modify the observed tracking?

**DOI:** 10.1186/s12966-019-0783-8

**Published:** 2019-02-15

**Authors:** Ingrid Marie Hovdenak, Tonje Holte Stea, Jos Twisk, Saskia Jacqueline te Velde, Knut-Inge Klepp, Elling Bere

**Affiliations:** 1 Department of Public Health, Sport and Nutrition, University of Agder, Kristiansand, Norway; 2 Department of Epidemiology and Biostatistics, the Amsterdam Public Health research institute, VU University Medical Center, Amsterdam, The Netherlands; 3 Department of Nutrition, Faculty of Medicine, University of Oslo, Oslo, Norway; 4 Department of Health and Inequalities & Centre for Evaluation of Public Health Measures, Norwegian Institute of Public Health, Oslo, Norway

**Keywords:** Tracking, Fruit, Vegetables, Unhealthy snacks, Dietary habits, Children, School fruit schemes, Intervention

## Abstract

**Background:**

The rationale for promoting increased consumption of fruit and vegetables (FV) at an early age is based on results from previous tracking-studies, indicating that dietary habits learned in childhood sustain into adulthood. Previous tracking studies have several limitations (e.g. low study sample, few repeated measurements and/or short a follow-up period). In addition, to our knowledge, no study has shown that a dietary intervention initiated in childhood affects tracking of dietary behaviour. The main objectives in this study were therefore to assess tracking of FV and unhealthy snacks in a large sample with multiple follow-up surveys over 15-years, and whether exposure to free school fruit for one school year modified tracking.

**Method:**

The longitudinal cohort-study, Fruit and Vegetables Make the Marks, included 38 randomly drawn schools in Norway; nine intervention schools received free fruit (or vegetable) in the school year 2001/2002 and 29 schools severed as control. The baseline sample included 1950 subjects, and 16–92% participated at five follow-up surveys (2002–2016). FV consumption and unhealthy snacks were measured by FFQ. Mixed models were applied to estimate overall tracking coefficients, and to assess whether the intervention modified tracking ((from baseline, from follow-up one (while intervention was running) and from follow-up two (after end of intervention)).

**Results:**

Overall tracking coefficients were 0.33 for fruit, 0.36 for vegetables and differed by sex for unhealthy snacks: 0.46 males and 0.39 for females (interaction *p* = 0.065). Most analyses showed no significant difference in tracking between the intervention group and control group. However, from follow-up one, tracking coefficients were different for unhealthy snacks, 0.46 vs. 0.38 (interaction *p* = 0.036), and from follow-up two for vegetables, 0.35 vs 0.48 (*p* = 0.036), in the intervention group and control group, respectively.

**Conclusion:**

Our results indicate low to moderate tracking of FV and unhealthy snacks from childhood to adulthood. We found little evidence that the free fruit intervention modified tracking of fruit, vegetables or unhealthy snacks. More research is needed on if or how we can influence the tracking of fruit, vegetables and unhealthy snacks consumption to improve public health.

**Electronic supplementary material:**

The online version of this article (10.1186/s12966-019-0783-8) contains supplementary material, which is available to authorized users.

## Background

As the health gain of consuming fruit and vegetables (FV) are well documented [1–5] and most western countries, including Norway, have a lower consumption than recommended [6–8], many public health initiatives have been launched to promote FV consumption. Notably, many of those initiatives have targeted children [9–12]. The rationale for promoting consumption of FV at an early age is often based on results from tracking-studies, which have shown that dietary habits to some extent sustain from childhood to adulthood [13]. By using tracking as an argument to implement interventions early, it is assumed that dietary interventions initiated in childhood will maintain to a significant extent later on in life i.e. that the newly gained intake levels will track throughout the rest of life. However, to our knowledge, no study has looked into the tracking of dietary behaviours after participating in an intervention nor whether interventions may influence the tracking of dietary behaviours.

Tracking can be defined as the maintenance of the relative position of a health related behaviour in rank at the group level, or the relative stability over a period of time [14]. Tracking studies which include fruit, vegetables, and unhealthy snacks (soft drinks, chocolate/sweets/crisps) as separate food groups have generally shown poor to moderate tracking [15–18]. Studies reporting dietary tracking have used longitudinal studies (e.g. health studies), usually including data from two or three timepoints and vary in the number of years covered and by sample size included per timepoint [13, 19]. Further, dietary methods used to assess tracking include; questionnaire items, FFQ, dietary history interviews, dietary recalls and diet diaries [19]. A wide range of statistical methods have been used to evaluate tracking; stability in rank, Kappa, odds ratio and correlation coefficients [13].

A systematic review reported that it was likely that the strength of dietary tracking was underestimated, due to methodological difficulties in quantifying habitual behaviour [13]. In general, tracking studies are difficult to compare due to difference in study design, methods of dietary assessment and use of statistical methods [14, 16, 19]. Furthermore, many studies only provide data from two or three time-points which limits the opportunity to gain knowledge into trajectories over time [19].

For the promotion of dietary habits, tracking provides insight into when dietary habits may persist over time, into which life stages people are responsive to interventions and can also be of use to improve the content of future interventions [17]. If an intervention is able to improve dietary behaviour while operating, it is interesting to examine if and how the behaviour (targeted by the intervention) continuous to track after the intervention period. Furthermore, as several dietary methods are used to examine dietary tracking, knowledge about how the use of dietary methods can affect the results are of importance.

In Norway, the Fruit and Vegetables Make the Marks (FVMM) research project provided one piece of fruit daily to children at elementary school for one year. Previous results from FVMM showed increased consumption of fruit in the intervention group while the intervention was operating [11], one [20] and three [21] and 14 (manuscript accepted for publication in AJCN), but not seven years after the intervention ended [22]. In addition, a significant reduction of consumption of unhealthy snacks was found among children with lower educated parents while the intervention was operating and seven years after the intervention ended [11, 22].

Thus, in the FVMM cohort, longitudinal data is available for fruit, vegetable and unhealthy snacks consumption, providing the opportunity to assess tracking with six repeated measurements over a period of 15 years. Moreover, the FVMM provides the opportunity to study whether tracking differs by dietary method. As mentioned, tracking studies vary in the methodologies used which limits comparability. In the current study FV intake was assessed by two different methods; i.e. a food frequency questionnaire (FFQ) and a 24-h recall (24-h). As the FFQ is a better measure for assessing habitual intake, we assume that this measure provides a more precise estimate while the 24-h recall is dependent on the intake at one day and as a result shows lower stability.

Therefore, the objectives of the present study are to 1) investigate the potential tracking of fruit, vegetable and snacks consumption from childhood to adulthood, 2) to investigate if exposure to one year of free school fruit at primary school modified tracking of fruit, vegetables and unhealthy snacks from childhood to adulthood and 3) to study if tracking coefficients differ by the dietary methods FFQ and 24-h recall.

## Materials and methods

### Design and study sample

The current study sample consists of participants from the FVMM cohort study. In 2001, 38 elementary schools from two counties (Hedmark and Telemark) in Norway were randomly drawn to participate in the FVMM study. Within one of the counties (Hedmark), nine schools were randomly selected as interventions schools and participated in the Norwegian School Fruit Scheme without parental payment; thus, receiving one piece of fruit or vegetable once every school day from October 2001 to June 2002 [21].

In September 2001, a baseline questionnaire survey was conducted. Follow-up surveys were conducted in May 2002, May 2003 (only 6th graders), May 2005, May 2009 and January–December 2016. The baseline questionnaire and three follow-up surveys (May 2002, May 2003 and May 2005) were conducted by trained research staff travelling to the respective schools. The fourth follow-up (May 2009) survey was sent by mail. At the fifth follow-up (2016), participants were contacted by phone and social media. A research profile was created for each of the four project workers to locate potential baseline participants. Verified participants were sent a private message with information about the project. The participants could answer the questionnaire by 1) receiving a link to the online questionnaire on Facebook or e-mail or 2) provide their answers on a phone interview.

The FVMM baseline cohort includes 1950 pupils; 966 girls and 984 boys, 585 were in the intervention group and 1365 in the control group. Estimated mean age was 11.8, 12.5, 12.0, 15.5, 19.5 and 26.5 years in 2001 (baseline), 2002, 2003, 2005, 2009 and 2016, respectively. A total of 1794 (92%), 918 (53%), 1602 (82%), 320 (16%) and 982 (50%) participated at the follow-up surveys in 2002, 2003, 2005, 2009 and 2016. For more information, see the consort flow chart [Additional file 1].

### Instruments

Two instruments were used to measure fruit and vegetable consumption; a 24-h recall and a food frequency questionnaire (FFQ), [Additional file 2]. The written 24-h recall was used to assess participants daily consumption of fruit and vegetables (portions/day). For the 24-h recalls conducted in 2001, 2002, 2003 and 2005, the pupils were requested to record their consumption the previous school day (surveys were conducted on weekdays, Tuesday through Friday). To help the pupils recall their intake, the day was divided into five periods: before school, at school, after school, at dinner and after dinner. The 24-h recall part of the questionnaire was read aloud to the participants in 2001 and 2002 (first follow-up) by a project worker. In 2009 and 2016, data included both weekdays and weekend days and participants were asked to report their FV consumption the previous day, and the day was separated into periods more applicable for adults: breakfast, lunch (between breakfast and dinner), dinner and supper (after dinner). At all timepoints, participants were also asked to record FV consumed in-between meals. Fruit and vegetable consumption was calculated separately (portions/day).

Additionally, usual fruit and vegetable consumption (times/week) was measured with four food frequency questions (FFQ). Unhealthy snacks consumption was measured by three FFQs (soda, candy, potato chips), which was merged into an unhealthy scale ranging from 0 to 30 times/week. The reply options for fruit, vegetable, and unhealthy snacks consumption were: Never, less than once a week, once a week, twice a week, three times a week, four times a week, five times a week, six times a week, every day, several times per day. The questions from the food frequency questions and the 24-h recall have previously been presented, and their validity and reliability have been reported for fruit and vegetable consumption among pupils in the 6th grade [23]. The FFQ questions used to measure consumption of unhealthy snacks have not been validated. A test-retest found that scores in the unhealthy snacks scale were significantly correlated, and mean values were not significantly different in two assessments 14 days apart [24].

At baseline, pupils reported their sex and their parents recorded their level of education (lower education: no college or university education or higher education: attended college or university).

### Data analysis

#### Descriptive and drop-out analysis

All statistical analysis was conducted using the statistical software package STATA 15.0 (StataCorp LLC, College station, TX, USA). All descriptive information is presented in Table 1, previous studies have explored differences in the FVMM dataset [20–22].

**Table 1:** Description of the sample at all timepoints by intervention and control

	Baseline (2001)			Follow-up 1 (2002)			Follow-up 2 (2003)*		
	Intervention	Control		Intervention	Control		Intervention	Control	
			*p*-value			*p*-value			*p*-value
N	585	1365		532	1262		293	625	
Gender (% female)	49	50	0.488	49	50	0.517	46	51	0.102
Parental education (% high)	48	39	< 0.001	48	39	0.001	46	40	0.115
	Mean ± SD	Mean SD		Mean ± SD	Mean ± SD		Mean ± SD	Mean ± SD	
Fruit (portions/day)	1.3 ± 1.5	1.5 ± 1.9	0.009	1.7 ± 1.6	1.2 ± 1.5	< 0.001	1.4 ± 1.4	1.2 ± 1.4	0.005
Fruit (times/week)	7.3 ± 4.4	7.6 ± 4.4	0.298	7.7 ± 4.1	7.2 ± 4.4	0.051	7.6 ± 4.2	7.4 ± 4.2	0.700
Vegetable (portions/day)	0.8 ± 1.1	0.9 ± 1.3	0.287	0.6 ± 1.0	0.6 ± 1.0	0.451	0.6 ± .09	0.6 ± 0.9	0.860
Vegetable (times/week)	6.5 ± 3.9	6.6 ± 3.8	0.763	7.1 ± 3.8	6.3 ± 3.9	< 0.001	6.6 ± 3.5	6.4 ± 3.6	0.280
Unhealthy Snacks (times/week)	6.5 ± 4.1	7.2 ± 7.7	0.001	6.3 ± 4.4	7.4 ± 5.1	< 0.001	6.0 ± 3.9	7.1 ± 5.0	< 0.001
	Follow-up 3 (2005)			Follow-up 4 (2009)			Follow-up 5 (2016)		
	Intervention	Control		Intervention	Control		Intervention	control	
			p-value			*p*-value			*p*-value
N	500	1101		112	208		297	685	
Gender (% female)	48	52	0.200	59	64	0.355	54	55	0.917
Parental education (% high)	48	40	0.004	53	46	0.229	54	48	0.141
	Mean ± SD	Mean ± SD		Mean ± SD	Mean ± SD		Mean ± SD	Mean ± SD	
Fruit (portions/day)	1.2 ± 1.4	1.2 ± 1.6	0.820	1.2 ± 1.4	1.0 ± 1.3	0.245	1.3 ± 1.3	1.2 ± 1.3	0.858
Fruit (times/week)	7.5 ± 4.3	7.0 ± 4.5	0.020	6.4 ± 4.3	6.0 ± 4.2	0.417	6.3 ± 3.6	6.3 ± 3.5	0.903
Vegetable (portions/day)	1.0 ± 1.3	0.9 ± 1.4	0.293	1.0 ± 1.1	1.0 ± 1.08	0.763	1.6 ± 1.4	1.5 ± 1.4	0.600
Vegetable (times/week)	6.9 ± 3.9	6.3 ± 3.7	< 0.001	6.6 ± 3.8	6.5 ± 3.9	0.825	7.8 ± 3.3	8.1 ± 3.4	0.301
Unhealthy snacks (times/week)	6.5 ± 5.2	7.2 ± 5.1	0.013	4.7 ± 3.4	5.4 ± 4.8	0.143	3.7 ± 2.6	4.1 ± 2.9	0.050

T-test for continuous variables. Chi square for categorical variables. *Follow-up 2 included only initially 6th graders (not 7th graders as they in May 2003 were in secondary elementary schools

To investigate the drop-out mechanism, two analyses were performed. Firstly, drop-out analysis to explore possible differences in fruit, vegetable and unhealthy snacks consumption at the previous measurement between drop-outs and respondents at all follow-ups (within groups) were done by independent t-test. Secondly, subjects with no-missing data (responded to all six measurements) were compared to subject with missing data (did not respond to 1 ≥ measurements) regarding weekly and daily consumption of fruit, vegetables and unhealthy snacks, gender and parental education over time.

#### Main analysis

Tracking was assessed by mixed model analyses, using all available longitudinal data, as suggested by Twisk [25] in which the baseline value was regressed on the outcome measured at the 5 follow-up measurements. Mixed model analyses must be used because the repeated observations within the subjects are highly correlated. The regression coefficient for baseline daily/weekly fruit, vegetable and unhealthy snacks consumption can be interpreted as the tracking coefficient, where values close to one indicate high tracking and values close to zero indicate low tracking. Further, the model allows for adjustment of covariates, both time-independent and time-dependent. The assumptions for linear mixed models were checked and met.

To analyse tracking of fruit, vegetables, and unhealthy snacks over a 15-year period the total sample was used with adjustment for the intervention. The analysis was repeated with the control group only, which gave the same results (analysis not shown). The models were further adjusted for parental education and/or sex and in addition, both parental education and sex was examined as a possible effect modifier and reported separately if the interaction was significant.

To investigate whether the intervention influenced tracking, assessment of tracking from respectively baseline, follow-up one and follow-up two were done by adding the interaction between the intervention and the initial measure to the model (baseline, follow-up one and two). Investigationsof tracking was done at several timepoints because; at baseline the control group had a higher consumption of fruit (24-h recall) than the intervention group. At follow-up one, the intervention was still operating, and the intervention group had higher consumption of fruit than the control group. At follow-up two, the intervention was over, however, only the initial 6th graders answered the questionnaire, as the initial 7th graders were in secondary high school in May 2003. Tracking analysis of vegetables by intervention was done, however previously published papers has indicated that school fruit schemes do not affect vegetable consumption [26]. Again, the analyses were adjusted for sex and parental education.

Tracking coefficients are not capable of giving information of predictability, therefore supplementary analysis was done with dichotomous outcomes of weekly/daily fruit and vegetables and unhealthy snacks. Subjects within the intervention and control group were categorized whether they were in the highest tertile of daily/weekly fruit and vegetables consumption and weekly unhealthy snacks consumption. Odds ratios were calculated for being in the highest tertile over time for subjects who were in the highest tertile at respectively baseline, follow-up one and two [27]. Odds ratios were calculated with logistic generalized estimation equation analysis [25].

To investigate if tracking differed by FFQ and 24-h recall the exact same analyses were applied to the longitudinal data derived from the two dietary methods.

We used the *p* < 0.05 to indicate statistical significant associations. For interaction terms *p* < 0.1 was used, as interaction terms are a multiplication of two variables which include measurement error [28].

## Results

### Description and analysis of missing data

Descriptive information of the study sample is shown in Table 1. Drop-out assessment between drop-outs and respondents at all follow-ups by the latter measurement [see Additional file 3], generally found that respondents had a higher mean consumption of fruit and vegetables and lower consumption of unhealthy snacks than drop-outs, both within the intervention and control group. However, there were more (at several timepoints) significant differences between drop-outs and respondents within the control group.

The mean consumption (over time) between responders who had had no-missing and 1 ≥ missing at follow-ups within the intervention and control group revealed no differences for weekly fruit consumption [see Additional file 4]. For weekly- and daily vegetable consumption, respondents with 1 ≥ missing had a lower mean consumption than no-missing within both groups. Regarding daily fruit consumption, responders with no-missing in the intervention group had a higher consumption, while in the control group respondents with 1 ≥ missing had a higher consumption. The mean consumption of snacks was higher among missing than no-missing in both groups. In both groups, a higher percentage of no-missing respondents were female, and their parents had a higher level of education compared to those who had 1 ≥ missing.

## Main results

The tracking coefficients were hardly affected by adjustments of sex and parental education; therefore, we only present the coefficients from the adjusted model.

### Overall tracking of fruit, vegetables and unhealthy snacks by dietary methods

#### FFQ

The adjusted tracking coefficient for weekly fruit and vegetable consumption, covering a period of 15-years was 0.33 (95% CI 0.29, 0.36) and 0.36 (95% CI 0.33, 0.40), Table 2. The tracking coefficient for snacks was significantly different for males and females 0.46 (95% CI 0.41, 0.51) vs 0.39 (95% CI 0.24, 0.44), respectively.

**Table 2:** Tracking of fruit, snacks and vegetables from baseline to follow-up 5 in the total sample

	Tracking**		
	Tracking coefficient	95% CI	*p*-value for interaction*
Fruit: times/week	0.33	0.29, 0.36	
Fruit portions/day			
Lower education	0.19	0.15, 0.22	0.006
Higher education	0.26	0.22, 0.30	
Vegetables: times/week	0.36	0.33, 0.40	
Vegetables: portions/day	0.14	0.11, 0.17	
Snacks: times/week			
Male	0.46	0.41, 0.51	0.065
Female	0.39	0.24, 0.44	
Longitudinal prediction of being in the highest tertile of intake***	OR	95% CI	
Fruit: times/week	2.64	2.26, 0.09	
Fruit: Portions/day	2.81	2.36, 3.35	
Vegetables: times/week			
Males	3.56	2.76, 4.61	0.004
Females	2.19	1.78, 2.70	
Vegetables: portions/day	1.78	1.51, 2.09	
Unhealthy Snacks: times/week	4.61	3.90, 5,44	

Adjusted for intervention, sex and parental education

*Results presented separately due to significant interaction *P* < 0.1

**Performed by Mixed Models

***Performed by GEE

#### 24-h recall

There was a significant (*p* = 0.006) interaction between baseline daily fruit consumption and parental education, as subjects with parents of lower education had a lower tracking (0.19 (95% CI 0.15, 0.22)) coefficient than subjects with higher education (0.26 (95% CI 0.22, 0.30)). The adjusted tracking coefficient for daily vegetable consumption was 0.14 (95% CI 0.11, 0.17).

#### Prediction of being in the highest tertile

Males had a significantly higher OR 3.56 (95% CI 2.76, 4,61) of remaining in the highest tertile of weekly vegetable consumption compared to females OR 2.19 (95% CI 1.78, 2.70). No other significant differences were found.

### Tracking of fruit, vegetables and unhealthy snacks by intervention and by dietary methods

#### FFQ

The tracking coefficient for snacks from follow-up one was significantly different in the intervention and control group, 0.46 (95% CI 0.39, 0.53) vs. 0.38 (95% CI 0.33, 0.42), respectively, Table 3. From follow-up two, the control group had a significantly higher tracking coefficient for weekly vegetables compared to the intervention group, 0.48 (95% CI 0.38, 0.59) vs. 0.35 (95% CI 0.28, 0.42). No other significant differences were found.

**Table 3:** Tracking and odds ratio of being in the highest tertile by intervention and control

	Tracking**				
	Intervention		Control		
	Tracking coefficient	95% CI	Tracking coefficient	95% CI	*p*-value for interaction*
Fruit: Times/week					
Baseline-follow-up 5	0.33	0.28, 0.39	0.33	0.29, 0.37	0.874
Follow-up 1 -follow-up 5	0.36	0.29, 0.43	0.39	0.34, 0.43	0.609
Follow-up 2 -follow-up 5	0.39	0.29, 0.49	0.37	0.31, 0.44	0.822
Vegetables: Times/week					
Baseline-follow-up 5	0.36	0.32, 0.40	0.37	0.32, 0.43	0.603
Follow-up 1 -follow-up 5	0.34	0.29, 0.38	0.39	0.32, 0.46	0.161
Follow-up 2 -follow-up 5	0.35	0.28, 0.42	0.48	0.38, 0.59	0.035
Unhealthy snacks: Times/week					
Baseline-follow-up 5	0.44	0.40, 0.48	0.40	0.33, 0.47	0.322
Follow-up 1 -follow-up 5	0.46	0.39, 0.53	0.38	0.33, 0.42	0.036
Follow-up 2 -follow-up 5	0.34	0.23, 0.44	0.38	0.32, 0.43	0.511
Fruit: Portions/day					
Baseline-follow-up 5	0.33	0.27, 0.39	0.18	0.15, 0.21	< 0.001
Follow-up 1 -follow-up 5	0.29	0.23, 0.35	0.19	0.15, 0.24	0.013
Follow-up 2 -follow-up 5	0.33	0.23, 0.43	0.26	0.18, 0.33	0.231
Vegetables: Portions/day					
Baseline-follow-up 5	0.13	0.10, 0.17	0.16	0.10, 0.22	0.493
Follow-up 1 -follow-up 5	0.17	0.19, 0.23	0.30	0.21, 0.38	0.013
Follow-up 2 -follow-up 5	0.27	0.18, 0.37	0.24	0.08, 0.38	0.662
Longitudinal prediction of being in the highest tertile of intake***	OR	95% CI	OR	95% CI	*p*-value for interaction*
Fruit: Times/week					
From baseline	2.50	1.90, 3.30	2.70	2.24, 3.27	0.650
From follow-up 1	2.31	1.65, 3.23	3.32	2.67, 4.14	0.074
From follow-up 2	2.71	1.70, 4.32	2.56	1.84, 3.58	0.533
Fruit: Portions/day					
From baseline	3.15	2.34, 4.23	2.65	2.13, 3.28	0.347
From follow-up 1	3.10	2.20, 4,33	2.15	1.61, 2.87	0.110
From follow-up 2	2.00	1.20, 3.32	2.61	1.76, 4.09	0.436
Snacks: Times/week					
From baseline	4.18	3.11, 5.62	4.81	3.93, 5.88	0.608
From follow-up 1	4.76	3.38, 6.70	4.46	3.51, 5.68	0.762
From follow-up 2	2.67	1.55, 4.60	5.90	4.12, 8.44	0.017
Vegetables: Times/week					
From baseline	3.08	2.32, 4.11	2.47	2.03, 3.01	0.209
From follow-up 1	3.23	2.32, 4.48	3.01	2.40, 3.79	0.740
From follow-up 2	3.84	2.34, 6.28	2.94	2.08, 4.16	0.389
Vegetables: Portions/day					
From baseline	1.82	1.37, 2.41	1.78	1.47, 2.17	0.941
From follow-up 1	2.41	1.69, 3.43	1.69	1.30, 2.19	0.112
From follow-up 2	1.75	1.09, 2.80	2.24	1.54, 3.26	0.418

All analysis was adjusted for sex and parental education

*interaction < 0.1 is considered significant

**Performed by Mixed Models

***Performed by GEE

#### 24-h recall

From baseline to follow-up five and from follow-up one to follow-up five, the daily tracking of fruit coefficient was significantly different for intervention- and control group 0.33 (95% CI 0.27, 0.39) vs 0.18 (95% CI 0.15, 0.21) and 0.29 (95% CI 0.23, 0.35) vs 0.19 (95% CI 0.15, 0.24), respectively, Table 3. From follow-up one, the control group had a significantly higher tracking coefficient for daily vegetables compared to the intervention group, 0.30 (95% CI 0.21, 0.38) vs. 0.17 (95% CI 0.19, 0.23). No other significant differences were found.

#### Prediction of being in the highest tertile

Prediction of being in the highest tertile for weekly fruit consumption from follow-up one to five was higher in the control group OR 3.32 (95% CI 2.67, 4.14) compared to the intervention group OR 2.31(95% CI 1.65, 3.23), Table 3. Further, the subjects in the control group (OR 5.90 (95% CI 4.12, 8.44)) had a higher predictability for being in the highest tertile of consumption of snacks compared to the intervention group (OR 2.67 (95% CI 1.55, 4.60)) from follow-up two. No other significant differences were found.

## Discussion

The current study presents tracking coefficients for fruit, vegetables and unhealthy snacks covering a 15-year period. To our knowledge, this is the first tracking study presenting separate tracking coefficients for fruit and unhealthy snacks after a dietary intervention.

### Overall tracking of fruit, vegetables and unhealthy snacks

Based on the FFQ, covering a 15-year period in the total sample, tracking coefficients of fruit and vegetables were 0.33 and 0.36, respectively, which is considered low to moderate and is in line with the literature [13]. Further, the tracking coefficient for unhealthy snacks were 0.46 for males and 0.40 for females, indicating that males had a more stable consumption over time. Predictability for weekly vegetable consumption was significantly higher for males that females, but predictability of fruit and unhealthy snacks consumption did not differ between the sexes.

Our analysis based on the 24-h recall found that subjects who had parents with higher education had a higher tracking coefficient of fruit than their counterparts. Although this result was not confirmed in the analysis based on the FFQ, it might indicate that stability of fruit consumption may be related to socioeconomic status, however this needs to be examined and explored in other studies.

The existing evidence of tracking of fruit, vegetables and unhealthy snacks in children and adolescents derives from studies with different design, various methods used and variation in number of years followed [13]. We have not been able to identify other studies using mixed models for assessing tracking of fruit, vegetables and unhealthy snacks which makes comparison difficult. Furthermore, no other studies have previously reported tracking of dietary habits after a dietary intervention period. A study by te Velde et al., however, used a similar statistical method (GEE) reported comparable tracking coefficients for fruit and vegetable over a 24-year period with six measurements [15]. Patterson et al. found poor tracking for vegetables, fair tracking for fruit and weak tracking for different snacks using Cohens’s weighted kappa over a 6-year period and the design of the study allowed for just two measurements [17]. The weak tracking coefficients in the latter study can be explained by the dietary method on which they based their analysis; one single 24-h recall, which is less suitable for assessing tracking. Further, Lien et al., analysed by stability and rank [16], and reported good tracking of fruit, vegetables and sweets/chocolate. Lake et al. used Pearson’s correlation coefficient and reported tracking for fruit and vegetables (combined) but found no correlation of sugary foods over a 20-year period [18]. The statistical methods and/or design used by Patterson, Lien and Lake has the disadvantage of only using two measurements as opposed to our study where the statistical method allowed for utilization of all available data. In our study, we only found a significant difference for tracking of snacks by gender, where boys had a higher tracking coefficient, as opposed to Lien et al. who found that girls had higher stability of consumption of sweets/chocolate and soft drinks [16].

### Tracking of fruit, vegetables and unhealthy snacks by intervention

Ideally, public health interventions and initiatives should have lifelong effects. The main argument used for initiation of interventions promoting increased FV consumption at an early age is that dietary habits learned in childhood “tracks” into adulthood. So far, no study has actually evaluated if tracking of diet over years has been modified by a dietary initiative. It is important to realize that the tracking coefficient concerns the relative position of a subject, so if everyone increases their intake the same amount (because of an intervention), the tracking coefficient will not change. It will only change if some subjects change more than others and when their relative position changes.

We found no significant difference between the intervention and the control group when analysing tracking of weekly fruit consumption. The tracking coefficient for unhealthy snacks was significantly higher in the intervention- compared to the control group from follow-up one (while the intervention was operating), but not from baseline and follow-up two (right after the intervention ended). Indicating that from follow-up one, the intervention group had a higher stability of unhealthy snacks compared to the control group. The subjects in the intervention group generally had a lower mean consumption of unhealthy snacks compared to the control group at all time-points (Table 1). Therefore, a higher tracking of unhealthy snacks shown in the present study indicates that the intervention group maintained a lower consumption over time. Further, the tracking coefficient for vegetables (times/week) was significantly higher in the control group from follow-up two, indicating a higher stability of consumption. However, the unadjusted consumption of vegetables (times/week) was lower in the control group compared to the intervention group at most timepoint, Table 1.

Predictability of remaining in the highest tertile of weekly fruit consumption from follow-up one was higher in the control group than the intervention group, but not from baseline and follow-up two. At follow-up one, the intervention was still operating and the subjects in the intervention group had a higher mean consumption of fruit. Therefore, it is likely that when the intervention ended, more subjects fell into the second and first tertile of consumption, thus lower predictability of remaining in the highest tertile. Further, within both groups and at all timepoints, subjects in the highest tertile at the initial measure had higher odds of remaining in the highest tertile over time, indicating that if you are a high consumer in childhood you are more likely to be a high consumer in adulthood (Table 3).

From follow-up two (just after the intervention ended) the participants in the control group had a significantly higher odds compared to the intervention group of remaining in the highest tertile of unhealthy snacks, but not from baseline and follow-up one, indicating that participants in the intervention group had a lower odds ratio of remaining in the highest tertile of unhealthy snacks.

Combined, the tracking- and prediction analyses used in the present study suggest that one year of school fruit provided without parental payment for 11.8-year-old children does not lead to different tracking coefficients of weekly fruit consumption from childhood to adulthood.

If the school fruit scheme actually modified tracking of fruit, we would expect lower tracking from baseline and higher tracking coefficients from follow-up one and two in the intervention group compared to the control group. A lower tracking coefficient from baseline would indicate a higher degree of fluctuation (within and between subject), preferably subjects with a low consumption increasing their intake over time. A higher tracking coefficient from follow-up one and two in the intervention group compared to control group would indicate that that the subjects to a higher degree maintained their high consumption over time. However, our prediction analysis suggests that if you are a high consumer in childhood you are also more likely to be a high consumer in adulthood.

On the other hand, we did observe significantly different tracking of unhealthy snacks between the groups from follow-up one; subjects in the intervention group had a higher tracking coefficient (and a lower consumption). These results may indicate that the school fruit scheme influenced tracking of unhealthy snacks. Yet, this was not sustained from follow-up two.

### Tracking coefficients by dietary methods

When interpreting the tracking coefficients, several considerations need to be kept in mind. Firstly, the magnitude of the tracking coefficient is dependent on the length of the time measurement(s) [29]. A high tracking coefficient over a short period of time may just be evidence of reliability of the used method, while a lower tracking coefficient over a long period of time, as found in this study, may be a stronger indication of tracking [29]. Secondly, tracking coefficients are influenced by measurement error. For instance, tracking coefficients for dietary consumption is lower than tracking of biological properties [30]. The reason for lower tracking of dietary consumption compared to i.e. biological properties is probably partly due to lower tracking, but also because it is difficult to measure dietary consumption, as eating behaviour can be highly variable both within and between days for an individual [30]. Hence, it is important to use a dietary measure which reflects habitual dietary consumption, i.e. FFQ, as used in this study, reflects habitual dietary (i.e. less measurement error) consumption better than a single 24-h recall. As mentioned, one single 24-h recall does not reflect usual dietary consumption at the individual level and may be more susceptible to daily variations and consequently provide low tracking coefficients. The current study confirmed that assumption. Still, the results of the 24-h recall are interesting and were thus presented, as it can be useful for other researchers to see how choice of dietary methods may influence results from tracking analysis.

### Strengths and limitations

The main strength of this study was that we assessed tracking over 15 years according to a dietary intervention, which has not been done before. Further, we used all six measurements and a suitable statistical test, providing knowledge into trajectories over time. We compared tracking coefficients derived from two dietary methods; FFQ and 24-h recall, where FFQ, is suitable for assessing tracking and gives an indication of habitual consumption [31]. Another strength of the present study is that both unhealthy and healthy dietary variables were studied, which provides a broader insight into dietary behaviour from childhood to adulthood.

Longitudinal studies are prone to loss of participants at follow-up, and this study is no exception. In 2009, the survey was sent to participants by regular mail. In addition, the participants had to send their completed survey back by regular mail. The delivery and return of the survey most likely led to the low participation in 2009. However, drop-out was much smaller for the other surveys. We performed two drop-out analyses which showed that drop-outs or subjects with one or several missing measurements, generally had a lower weekly consumption of fruit and vegetables and higher consumption of unhealthy snacks. Further, girls and subjects with higher educated parents were better represented at the follow-ups. The rather large drop-out may have affected the results, however, we used mixed models for repeated measurements, which is an analysis that handles data with missing observations. Although we adjusted for sex and parental education, there might be other confounders e.g. total energy consumed which we did not have the opportunity to control for. The population in the FVMM is not a representative sample of the Norwegian population regarding parental education. In 1999, 28,5% of the population in Norway aged 34–44 years had higher education, which is lower than observed in the FVMM study [32].

The main challenge of estimating tracking coefficients for eating behaviour lies in measurement challenges, as eating behaviour can be highly variable both within and between days for an individual. Therefore, we don’t know how much of the low to moderate tracking coefficient may be explained by measurement error. In addition, all methods for collecting self-reported dietary data have well-known limitations [33].

Another limitation of this study is the Norwegian School Fruit Scheme (NSFS). The NSFS is a paid subscription program providing daily fruit to pupils at school, while the FVMM intervention was operating. Initially, schools could decide if they wanted to participate in the program. At participating schools’ parents could decide if they wanted to subscribe. All 29 FVMM control schools in the were given the opportunity to participate in the NSFS. Nine control schools participated, and 20 control schools declined to participate in the programme in the school year 2001/2002. In the nine control schools participating, there were both subscribers and non-subscribers (41% of the pupils subscribed).

Another limitation of the present study was the use of a non-validated measure for unhealthy snacks. Additionally, unhealthy snacks are a combined measure, which included both information about soda, crisps and candy/chocolate consumption. Therefore, we were not able to identify the contribution of the different snacks items to the tracking coefficient and how the foods would track separately.

## Conclusions

Our results indicate low to moderate tracking of fruit, vegetables and unhealthy snacks covering a 15-year period. These findings suggest that these dietary habits persist from childhood to adulthood, but are not yet stabilized in childhood, indicating that the habits are modifiable. We found little evidence that providing school fruit for one year in primary school led to changes in tracking of fruit, vegetables or unhealthy snacks. More research is needed on if or how we can influence the tracking of fruit, vegetables and unhealthy snacks consumption to improve public health: I.e. is it possible to increase children’s consumption of fruits and vegetables and does this increased level track into adulthood? The current study indicates that FFQ reflects habitual dietary consumption better than one single 24-h recall and is therefore more appropriate when assessing tracking.

## Additional files

Additional file 1: CONSORT flow chart (word-file). (DOC 53 kb)

Additional file 2: First page of both questionnaires (24 h-recall and FFQ) (PDF-file). (PDF 7 kb)

Additional file 3: Table: Difference between drop-outs and respondents by group (word-file). (DOCX 19 kb)

Additional file 4: Table: Overall difference mean/percentage between 1  ≥ missing and no missing within the intervention and control group (word-file). (DOCX 16 kb)

## Abbreviations

FV: Fruit and vegetables
FVMM: Fruit and Vegetables Make the Marks
GEE: Generalized estimation equation analysis
NSFS: Norwegian School Fruit Scheme
SES: Socioeconomic status
