# Supplementary material for: Tracking of fruit, vegetables and unhealthy snacks consumption from childhood to adulthood (15 year period): does exposure to a free school fruit programme modify the observed tracking?
Source: Int J Behav Nutr Phys Act. 2019 Feb 15;16:22. doi: 10.1186/s12966-019-0783-8 (PMC6377717; doi:10.1186/s12966-019-0783-8)
Supplement: Supplementary file 1 — CONSORT flow chart (word-file). (DOC 53 kb) [file 12966_2019_783_MOESM1_ESM.doc]

**
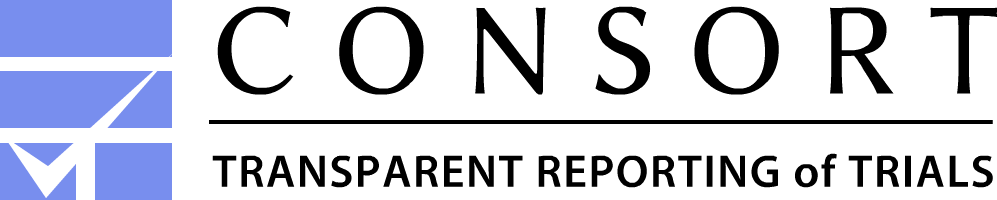
**

**CONSORT 2010 Flow Diagram**

**Allocation**

**Analysis**

**Follow-Up**

**Enrollment**

Assessed for eligibility: 48 elementary schools

24 in the county of Hedmark; Norway

24 in the county of Telemark; Norway

10 schools declined to participate: 5 from each county

Analysed from:

Baseline (n=585)

Follow-up 1 (n=532)

Follow-up 2 (n=293)

Follow-up 3 (n=500)

Follow-up 4 (n=112)

Follow-up 5 (n=297)

All data were analyzed

Lost to follow-up 1 (n=53)

Lost to follow-up 2 (n=292)

Lost to follow-up 3 (n=85)

Lost to follow-up 4 (n=473)

Lost to follow-up 5 (n=288)

Due to:

1) unable to find contact information

2) declined to participate

9 schools allocated to intervention from Hedmark county (n=585)

Lost to follow-up 1 (n=103)

Lost to follow-up 2 (n=740)

Lost to follow-up 3 (n=255)

Lost to follow-up 4 (n=1157)

Lost to follow-up 5 (n=680)

Due to:

1) unable to find contact information

2) declined to participate

29 schools allocated to control, 19 from Telemark county and 10 from Hedmark county (n=1365)

Analysed from:

Baseline (n=1365)

Follow-up 1 (n=1262)

Follow-up 2 (n=625)

Follow-up 3 (n=1101)

Follow-up 4 (n=208)

Follow-up 5 (n=685)

All data were analyzed

A total of 38 elementary schools: 19 from Hedmark and 19 from Telemark county

Randomization to intervention: 9 of 19 schools from Hedmark county
