# Supplementary material for: Tracking of fruit, vegetables and unhealthy snacks consumption from childhood to adulthood (15 year period): does exposure to a free school fruit programme modify the observed tracking?
Source: Int J Behav Nutr Phys Act. 2019 Feb 15;16:22. doi: 10.1186/s12966-019-0783-8 (PMC6377717; doi:10.1186/s12966-019-0783-8)
Supplement: Supplementary file 2 — First page of both questionnaires (24 h-recall and FFQ) (PDF-file). (PDF 7 kb) [file 12966_2019_783_MOESM2_ESM.pdf]

Tenk tilbake til i går tidlig

Frokost

1. Spiste du frokost i går tidlig?

☐ Ja ☐ Nei

2. Spiste du frukt eller bær før du begynte på skolen i går tidlig?

☐ Ja ☐ Nei

Hvis ja, skriv ned hva slags og hvor mye **frukt** og **bær** du spiste her:

3. Spiste du grønnsaker før du begynte på skolen i går tidlig?

☐ Ja ☐ Nei

Hvis ja, skriv ned hva slags og hvor mye **grønnsake**r du spiste her:

4. Drakk du juice før du begynte på skolen i går tidlig?

☐ Ja ☐ Nei

Hvis ja, skriv ned hva slags og hvor mye **juice** du drakk her:

Del C

Hva spiser du vanligvis?
Når du fyller ut disse spørsmålene skal du tenke på hva du vanligvis spiser/drikker. Tenk gjerne på hva du har spist/drukket de siste 3 månedene. Tenk på både hva du spiser hjemme, på skolen og i fritiden. Kryss av i den ruten du føler passer best for deg.

HUSK: Kun ett kryss for hvert spørsmål!

1. Hvor ofte spiser du potet?

- ☐ Aldri
- ☐ Sjeldnere enn 1 gang i uken
- ☐ 1 gang i uken
- ☐ 2 ganger i uken
- ☐ 3 ganger i uken
- ☐ 4 ganger i uken
- ☐ 5 ganger i uken
- ☐ 6 ganger i uken
- ☐ Hver dag
- ☐ Flere ganger hver dag

2. Hvor ofte spiser du grønnsaker til middag?

- ☐ Aldri
- ☐ Sjeldnere enn 1 gang i uken
- ☐ 1 gang i uken
- ☐ 2 ganger i uken
- ☐ 3 ganger i uken
- ☐ 4 ganger i uken
- ☐ 5 ganger i uken
- ☐ 6 ganger i uken
- ☐ Hver dag
- ☐ Flere ganger hver dag

3. Hvor ofte spiser du grønnsaker på brødskivene? (f.eks. agurk, tomat, paprika)

- ☐ Aldri
- ☐ Sjeldnere enn 1 gang i uken
- ☐ 1 gang i uken
- ☐ 2 ganger i uken
- ☐ 3 ganger i uken
- ☐ 4 ganger i uken
- ☐ 5 ganger i uken
- ☐ 6 ganger i uken
- ☐ Hver dag
- ☐ Flere ganger hver dag

4. Hvor ofte spiser du andre grønnsaker?
(f.eks. gulrot til skolematen)

- ☐ Aldri
- ☐ Sjeldnere enn 1 gang i uken
- ☐ 1 gang i uken
- ☐ 2 ganger i uken
- ☐ 3 ganger i uken
- ☐ 4 ganger i uken
- ☐ 5 ganger i uken
- ☐ 6 ganger i uken
- ☐ Hver dag
- ☐ Flere ganger hver dag

5. Hvor ofte spiser du eple, appelsin, pære og banan?

- ☐ Aldri
- ☐ Sjeldnere enn 1 gang i uken
- ☐ 1 gang i uken
- ☐ 2 ganger i uken
- ☐ 3 ganger i uken
- ☐ 4 ganger i uken
- ☐ 5 ganger i uken
- ☐ 6 ganger i uken
- ☐ Hver dag
- ☐ Flere ganger hver dag
