# Supplementary material for: Tracking of fruit, vegetables and unhealthy snacks consumption from childhood to adulthood (15 year period): does exposure to a free school fruit programme modify the observed tracking?
Source: Int J Behav Nutr Phys Act. 2019 Feb 15;16:22. doi: 10.1186/s12966-019-0783-8 (PMC6377717; doi:10.1186/s12966-019-0783-8)
Supplement: Supplementary file 3 — Table: Difference between drop-outs and respondents by group (word-file). (DOCX 19 kb) [file 12966_2019_783_MOESM3_ESM.docx]

|  | **Intervention** | | | **Control** | | |
| --- | --- | --- | --- | --- | --- | --- |
|  | Missing | Respondent |  | Missing | Respondent |  |
|  | Mean ± SD | Mean ± SD | p-value | Mean ± SD | Mean ± SD | p-value |
| **Fruit: Times/week** |  |  |  |  |  |  |
| Follow-up 1 | 6.9±5.3 | 7.4±4.3 | 0.452 | 7.8±4.4 | 7.5±4.4 | 0.601 |
| Follow-up 2 | 7.5±4.2 | 7.8±4.0 | 0.436 | 7.0±4.4 | 7.6±4.4 | 0.024 |
| Follow-up 3 | 8.5±5.7 | 7.4±4.0 | 0.222 | 7.4±4.4 | 7.4±4.2 | 0.883 |
| Follow-up 4 | 7.5±4.3 | 7.8±4.4 | 0.486 | 6.7±4.5 | 7.1±4.6 | 0.592 |
| Follow-up 5 | 6.0±4.0 | 6.6±4.4 | 0.520 | 5.0±3.5 | 6.5±4.4 | 0.015 |
| **Fruit: Portions/day** |  |  |  |  |  |  |
| Follow-up 1 | 1.2±1.7 | 1.3±1.5 | 0.372 | 1.5±1.7 | 1.6±1.9 | 0.687 |
| Follow-up 2 | 1.8±1.7 | 1.7±1.4 | 0.919 | 1.1±1.5 | 1.3±1.5 | 0.080 |
| Follow-up 3 | 1.1±1.5 | 1.4±1.4 | 0.312 | 1.4±1.5 | 1.2±1.4 | 0.377 |
| Follow-up 4 | 1.1±1.4 | 1.5±1.5 | 0.033 | 1.2±1.6 | 1.4±1.8 | 0.095 |
| Follow-up 5 | 0.8±1.0 | 1.3±1.6 | 0.129 | 0.7±1.2 | 1.2±1.4 | 0.025 |
| **Vegetables: Times/week** |  |  |  |  |  |  |
| Follow-up 1 | 5.0±3.8 | 6.6±3.9 | 0.003 | 6.3±4.0 | 6.6±3.9 | 0.438 |
| Follow-up 2 | 6.8±3.8 | 7.4±3.6 | 0.066 | 5.9±3.9 | 6.7±6.3 | 0.001 |
| Follow-up 3 | 5.8±3.7 | 6.7±3.4 | 0.158 | 6.2±3.5 | 6.4±3.6 | 0.639 |
| Follow-up 4 | 6.8±3.9 | 7.6±3.8 | 0.061 | 6.1±3.7 | 6.8±4.0 | 0.024 |
| Follow-up 5 | 6.5±3.7 | 6.6±3.8 | 0.878 | 5.8±3.7 | 6.8±3.9 | 0.050 |
| **Vegetables: Portions/day** |  |  |  |  |  |  |
| Follow-up 1 | 0.8±1.1 | 0.9±1.1 | 0.698 | 0.6±0.9 | 0.9±1.3 | 0.030 |
| Follow-up 2 | 0.6±1.1 | 0.6±0.9 | 0.643 | 0.6±0.9 | 0.7±1.0 | 0.083 |
| Follow-up 3 | 0.6±0.9 | 0.6±0.9 | 0.770 | 0.5±0.9 | 0.6±0.9 | 0.377 |
| Follow-up 4 | 0.9±1.3 | 1.1±1.4 | 0.132 | 0.9±1.4 | 1.0±1.5 | 0.080 |
| Follow-up 5 | 1.2±1.1 | 1.0±1.1 | 0.642 | 0.8±1.0 | 1.1±1.0 | 0.042 |
| **Unhealthy snacks: Times/week** |  |  |  |  |  |  |
| Follow-up 1 | 7.6±4.9 | 6.5±4.0 | 0.066 | 8.6±5.3 | 7.1±4.6 | 0.004 |
| Follow-up 2 | 6.8±4.5 | 5.9±4.2 | 0.021 | 7.9±5.2 | 6.8±4.8 | <0.001 |
| Follow-up 3 | 5.3±4.5 | 6.0±3.8 | 0.336 | 7.2±4.8 | 7.1±5.0 | 0.791 |
| Follow-up 4 | 6.7±5.4 | 5.5±4.5 | 0.042 | 7.4±5.2 | 6.1±4.6 | 0.003 |
| Follow-up 5 | 5.5±4.3 | 4.5±3.0 | 0.146 | 6.5±5.1 | 4.9±3.5 | 0.006 |

Additional file 3 Difference between drop-outs and respondents by group

*t-test for continuous variables and chi-square for categorical variables.
