# Supplementary material for: Tracking of fruit, vegetables and unhealthy snacks consumption from childhood to adulthood (15 year period): does exposure to a free school fruit programme modify the observed tracking?
Source: Int J Behav Nutr Phys Act. 2019 Feb 15;16:22. doi: 10.1186/s12966-019-0783-8 (PMC6377717; doi:10.1186/s12966-019-0783-8)
Supplement: Supplementary file 4 — Table: Overall difference mean/percentage between 1 ≥ missing and no missing within the intervention and control group (word-file). (DOCX 16 kb) [file 12966_2019_783_MOESM4_ESM.docx]

|  | Intervention | | | Control | | |
| --- | --- | --- | --- | --- | --- | --- |
|  | No missing | Missing |  | No missing | Missing |  |
|  | Mean ± SD | Mean ± SD | p-value | Mean ± SD | Mean ± SD | p-value |
| Fruit: Times/week | 7.3±4.7 | 7.3±4.3 | 0.894 | 6.9±3.9 | 7.1±4.3 | 0.304 |
| Fruit: Portions/day | 1.5±1.5 | 1.4±1.5 | 0.131 | 1.1±1.3 | 1.3±1.7 | 0.038 |
| Vegetables: Times/week | 7.4±3.8 | 6.9±3.7 | 0.083 | 7.1±3.7 | 6.6±3.8 | 0.004 |
| Vegetables: Portions/day | 1.0±1.3 | 0.9±1.2 | 0.041 | 1.1±1.2 | 0.9±1.2 | 0.004 |
| Unhealthy snacks: Times/week | 5.1±3.7 | 6.0±4.4 | 0.002 | 5.8±4.1 | 6.9±4.9 | <0.001 |
|  | % | % |  | % | % |  |
| Gender (% female) | 59 | 48 | 0.002 | 63 | 50 | <0.001 |
| Parental education (%high) | 59 | 47 | 0.002 | 54 | 38 | <0.001 |

Additional file 4 Overall difference mean/percentage between 1< missing and no missing within the intervention and control group

*t-test for continuous variables, chi-square for categorical variables.
